# REHABILITATION WITH INTENSIVE ATTENTION TRAINING EARLY AFTER ACQUIRED BRAIN INJURY PROMOTES BETTER LONG-TERM STATUS ON HEALTH-RELATED QUALITY OF LIFE, DAILY ACTIVITIES, WORK ABILITY AND RETURN TO WORK

**DOI:** 10.2340/jrm.v56.5308

**Published:** 2024-01-12

**Authors:** Gabriela MARKOVIC, Aniko BARTFAI, Marie-Louise SCHULT, Jan EKHOLM

**Affiliations:** 1 Department of Clinical Sciences, Karolinska Institutet, Danderyd University Hospital, Stockholm; 2 Division of Rehabilitation Medicine, Danderyd University Hospital, Stockholm, Sweden

**Keywords:** acquired brain injury, cognitive rehabilitation, early rehabilitation, attention dysfunction, attention process training, activity and participation

## Abstract

**Objective:**

To describe long-term effects on activity, participation, and quality of life (*i*) at different post-injury starting time points of attention training and (*ii*) of two different types of rehabilitation with attention training in patients after stroke or traumatic brain injury; and to describe their functioning level.

**Design:**

2 years after rehabilitation intervention, comparisons were made in one cohort receiving attention training subacute (< 4 months) or post-acute (4–12 months) and in one cohort with two different training methods, a process-based and an activity-based method respectively.

**Patients:**

100 patients were recruited from our earlier RCT study. They had mild to moderate stroke or traumatic brain injury with relatively limited symptomatology, and all had moderate to severe attention impairment.

**Methods:**

A questionnaire-based interview: EuroQol 5 dimensions, Occupational Gaps Questionnaire, Work Ability Index, self-assessed work status, self-reported employment conditions, sick leave, and experienced cognitive limitations in work performance.

**Results:**

An advantage for patients receiving subacute attention training regarding daily activities, work ability and returning to work.

**Conclusion:**

The results indicate that subacute rehabilitation with attention training (< 4 months) is preferable compared to post-acute intervention (4–12 months). There were only minor differences between the training methods.

LAY ABSTRACT

Previously, in the context of interdisciplinary rehabilitation, we have subjected patients with stroke or traumatic brain injury to additional intensive rehabilitation of attention using two different training methods, a process-based and an activity-based method. The specific attention training commenced either during the first four months after injury or between four and 12 months after injury. In the current study, we report data collected by a telephone interview two years later regarding quality of life and possible limitations in activities of daily life and work. The results show an advantage for patients receiving early rehabilitation regarding daily activities, capacity for work and return to work. Further, there were some minor advantages for patients in the early group, receiving process-based training. Still, the two methods had no significant outcome differences after two years. Thus, the importance of early rehabilitation needs to be considered in organizing rehabilitation services.

Attention difficulties are common cognitive sequelae of acquired brain injury (ABI) (1, 2). Attention supports other cognitive functions and, as such, it is a core component of cognitive skills underlying human activities. Small changes in attention might significantly impact a person’s daily life by affecting learning skills, daily functioning, and work (3, 4).

There are a variety of approaches for rehabilitation of attention impairments depending on injury severity and the specific nature of the attention impairment, with interventions that are either restorative, designed to improve underlying cognitive processes, or compensatory, improving performance and allocating attention resources (5–7). One of these approaches is systematic, hierarchical training with attention process training (APT) (8). This method targets the different aspects of attention, i.e., focused, sustained, selective, alternating, and divided into tasks of increasing difficulty and complexity. APT is a successful method post-ABI for adults (9–11) and children (12). APT is recommended in several guidelines (6, 7) and is considered an evidence-based method in the chronic stage after ABI. Most studies report these favourable effects from a post-intervention perspective. In contrast, long-term studies are scarce (4, 13), with only 2 studies focusing on the long-term effect of APT intervention in terms of strategy use (14) and maintenance of functional level (15). Therefore, the current study focuses on long-term aspects.

Activity-based attention training (ABAT) is a performance-skills and metacognitive strategy training emphasizing the importance of conscious cognitive processes in initiating, performing, and controlling attention-demanding activities, such as cooking and studying (5, 10, 16, 17).

Previously, we conducted a series of RCT studies investigating the short-term effects of attention training within the first year after ABI (18), comparing the effects of APT and ABAT in combination with an interdisciplinary inpatient and outpatient rehabilitation programme in 2 cohorts (18–20). Patients from the 2 largest diagnostic groups with ABI, stroke, and traumatic brain injury (TBI) were included in the study, following the same inclusion and exclusion criteria. The results of this short-term rehabilitation suggested an advantage for patients receiving APT (20–22)**.** The current study examines long-term effects of the choice of rehabilitation intervention.

The definition of time intervals for rehabilitation of subacute ABI, compared with chronic ABI, varies, but the first year after ABI is usually considered an early stage (23). However, the path of spontaneous recovery of cognitive functions after ABI is described as non-linear during that timeframe, with different physiological mechanisms and a steeper curve during the first 3–4 months (24). Consequently, data were collected from 2 cohorts (18) since, during the past decade, several studies have reported positive effects of subacute cognitive rehabilitation (< 3 months) after ABI (25–32). These studies reported substantial changes during inpatient rehabilitation. Although effect sizes subsided over time, significant improvements were maintained up to 3 months after discharge. Thus, the choice of the starting time-point for attention-improving interventions is relevant.

People of working age with stroke or TBI are expected to live with a lifelong disability affecting both well-being and health-related quality of life (HRQoL). Active coping strategies (33) and return to work (RTW) have proved to be of importance for well-being after ABI (34–36), while cognitive impairment is associated with worse HRQoL (37) and attention dysfunction specifically (38–41) more likely results in failure to RTW. The possibility of RTW (42) depends not only on medical and psychological pre-and post-injury factors (43), but also on workplace-related circumstances (44). Several studies have highlighted the importance of investigating the long-term effects of cognitive rehabilitation (4, 36, 45, 46) along with the need to measure these changes in terms of changes in activity and participation (47), since those measures are assumed to reflect closer real-life changes in, for example, personal independence and work situation (48). With this background, the current study examines the patients’ working ability and RTW 2 years after rehabilitation.

This study describes the activity status, participation, perceived work ability, and HRQoL in participants (18) receiving intensive attention training within the first year post-ABI. The study aimed to compare the long-term effects of: (*i*) subacute start of attention training with post-acute start; and (*ii*) 2 different attention interventions, process-based and activity-based. Based on our previous demonstration of an advantage of APT training within 4 months post-injury (21, 49) and a positive effect on work-performance within 4–12 months post-injury (20), one of the current study hypotheses was that the long-term effectiveness of APT training would be greater than that of the ABAT form of attention training in patients studied 2 years after the initial training.

## METHODS

### Study design

Participants from the previous RCT study (ClinicalTrials.gov.trial registration NCT02091453) (18, 50) were recruited 2 years after the initial rehabilitation intervention. Comparisons were made in 1 cohort that had undergone attention training either subacute (SA) (< 4 months post-ABI) or post-acute (PA) (4–12 months post-ABI) and in a second cohort that had undergone 1 of 2 different training methods, a process-based method (APT) and an activity-based method (ABAT).

### Participants

The participants had had either a mild-to-moderate stroke or TBI with relatively homogenous symptomatology, no aphasia, psychiatric symptoms, or neglect. Satisfactory levels of logical reasoning, memory, and fine motor functions were required to participate in the APT programme. Attention impairment was moderate to severe, measured by the APT test (8) preceding the attention training. Demographic characteristics are shown in Table I. Injury-related characteristics (lesion localization and distribution) are shown in Table II: a rehabilitation physician and a neuropsychologist classified lesion distribution and related features.

**Table I:** Sociodemographic characteristics at follow-up (median = 22 months post-intervention; 95% confidence interval (95% CI) 21–24 months) for participants (*n* = 100) in interdisciplinary rehabilitation after acquired brain injury

Variable	Total sample
Age, years, mean (SD)/median	49 (10)/52
Age range, *n* (%)	
19–29 years	6
30–49 years	36
50–64 years	58
Sex, female, *n*	49
Marital status, *n*	
Married/co-habitant	76
Single	22
With parents	2
Education^a^, *n*	
≤ 12 years	31
13–15 years	44
> 16 years	25
Employment (time of injury), *n*	
Working or studying	81
Not working	19

a Completed years of education, from elementary school to higher education.

SD: standard deviation.

**Table II:** Lesion distribution at follow-up of participants (*n* = 100) in interdisciplinary rehabilitation after acquired brain injury

Variable	Total sample
Aetiology, *n*	
Stroke^a^	79
Traumatic brain injury^b^	21
Lesion side, *n* (%)	
Left/right hemisphere	42/31
Bilateral	27
Lesion distribution, *n* (%)	
Focal/multifocal (≥ 2)	47/53
Lesion localisation, *n* (%)	
Anterior	25 (29)
Posterior	16 (19)
Subcortical	35 (41)
Global	9 (11)

a Of which strokes 61% were thrombosis, 27% haemorrhage, 10% subarachnoid haemorrhage, and 2% thrombosis and haemorrhage.

b Eight participants had haematoma, 4 had contusions, and 9 had both haematoma and contusions. Traumatic brain injury was a result of traffic accidents (*n* = 9), falls (*n* = 8), sports (*n* = 3) and assault (*n* = 1).

### Procedure

An experienced research nurse, who was unfamiliar with the participants and blinded to intervention, contacted the participants, and performed data collection by telephone. All participants (*n =* 120) from the previous RCT (18) were contacted by letter and a subsequent telephone call. Of these, 16 did not respond, 3 did not have a contact address, and 1 was deceased; therefore, the final number of respondents was 100. After signing informed consent, the participants received the questionnaires by post, and the research nurse interviewed them within 1 week.

### Outcome assessments

The current study selected outcome measures (51) guided by a philosophy that outcome research in ABI should focus on function and participation in daily life. This study describes the status of the participants in relation to a healthy reference group according to each measure’s standard outcome.

*Health-related quality of life EuroQol-5 dimensions questionnaire (3L)*. EQ-5D, a generic, patient-reported HRQoL instrument, consists of 5 dimensions (mobility, self-care, usual activities, pain/discomfort, and anxiety/depression) and 3 severity levels (no problems, moderate problems, severe problems) (52). A single-index value can be derived for each dimension. The dimensions are converted into an index value (−0.594 and + 1.000) (53), anchored at 1 (total health) and 0 (dead). Swedish normative health index is 0.898 for men and 0.886 for women (54). The EQ-5D is a valid measure of quality of life (QoL) after stroke (55).

*Occupational Gaps Questionnaire.* The OGQ, version 1.1, measures perceived participation in everyday occupations (56). An occupational gap (OG) occurs when the individual: (*i*) does not perform an activity that they want to (OG 1); or (*ii*) performs an activity that they do not want to (OG 2). This study considers only OG 1. OGs were examined for 28 activities, including 8 instrumental ADLs, 6 social activities, 10 leisure activities and 4 work-related activities. Higher scores correspond to higher restrictions. The scale is dichotomous, distinguishing situations with no OG from conditions with OGs. The results are presented regarding the distribution of OGs in relation to a healthy reference group (56).

*Work ability.* The Work Ability Index (WAI) (57) measures work ability in 7 dimensions: current work ability (WAS) compared with lifetime best (score 0–10), work ability concerning job demands (score 2–10), number of medical diagnoses (score 1–7), impaired work performance (score 1–6), sickness absence in the last 12 months (score 1–5), expected work ability in the forthcoming 2 years (score 1–7), and mental resources (score 1–4). Scores are summed with (range 7–49) and without the diagnosis list (range 6–42). High numbers indicate better self-reported work ability. The number of diagnoses was based on medical records at inclusion. Results are presented in relation to a healthy reference group (58). The total WAI score can be grouped into 4 classes of work ability: 1: “poor” (need to restore); 2: “moderate” (need to improve); 3: “good” (need to support); and 4: “excellent” (need to maintain) (59). Self-rated work ability score (WAS) is based on the first question: “Current work ability compared with the lifetime best” (0 = complete work disability, 10 = best work ability). Previous studies demonstrate a strong association between WAS and the complete WAI. Therefore, WAS has been recommended as a simple, reliable indicator of work ability (60).

*Self-reported employment conditions, sick leave, and experienced cognitive limitations in work performance.* A structured questionnaire developed by the research group covered self-reported employment conditions, sick leave, and experienced cognitive limitations in work performance. Eight questions concerning employment conditions could be answered with “yes” or “no”. Nine options were presented for the nature and extent of the actual work situation and sickness allowance/sickness compensation (0, 1/4, ½, ¾, or total compensation). The participants could select more than 1 option. Questions (*n* = 17) concerning experienced limitations in work performance due to the ABI could be answered with “yes”, “sometimes”, and “no”. The questions focused on cognitive and behavioural difficulties in work due to attention dysfunction, such as concentration demands, understanding and completing work tasks, forgetfulness, error-proneness, problems initiating and structuring work tasks, and the need for technical and emotional support.

### Previous interdisciplinary rehabilitation and attention training

All subjects in the current study had participated in intensive (6 h/day, 4–5 day/week for 8–12 weeks) interdisciplinary rehabilitation, which was completed 2 years prior to the start of the current study. The rehabilitation was provided by rehabilitation physician, nurse, physiotherapist, occupational therapist, neuropsychologist, speech and language therapist, and social worker. Cognitive interventions were based on the work of the Cognitive Rehabilitation Task Force (CRTF) of the American Congress of Rehabilitation Medicine (10, 61). In addition to the regular rehabilitation, the participants received a total of 20 h of attention training, 3–5 times/week, for 5–6 weeks. The attention training was randomized into APT or ABAT.

The APT (8) is a process-oriented, theoretically and hierarchically based, individualized attention-training programme considered a “practice standard” treatment for attention deficits after brain injury (10, 61). It comprises repetitive exercises with increased difficulty and meta-cognitive strategy training for improved and more flexible use of strategies in daily life (generalization), insight, and motivation. The APT is a direct structured neuropsychological intervention. A neuropsychologist performs the training session individually (45–90 min/session), improving performance on training tasks and immediate measures of global attention (10).

The ABAT is an occupational therapy intervention comparable to the Cognitive Orientation to Occupational Performance (CO-OP) (62, 63) aiming at functional skills training on activity level and metacognitive strategies to improve performance on trained tasks. An occupational therapist conducted ABAT involving attention-demanding everyday activities in personal care, household activities, work, leisure, and social activities (60–120 min/session) (20).

### Statistical analysis

Pearson χ^2^ test was used to analyse sex, marital status, education, and injury-related data. A parametric *t*-test was used to compare groups on age, timing and length of intervention, the timing of follow-up, results of psychometric testing, and level of attention dysfunction. Descriptive statistics (frequencies, mean, median, standard deviations, percentiles, and confidence intervals) were calculated for the telephone interview questionnaires. Group comparison was analysed with the Pearson χ^2^ test. Statistical differences between groups and subgroups were analysed for self-rated health expressed in EQ-5D dimensions (Pearson χ^2^) and EQ-5D index (Mann–Whitney *U* test).

For the OGQ, this study presents data on OG 1 (i.e., “does not perform an activity that he/she wants to”). The proportion of reported OGs in the study population was compared with a Swedish age-matched reference population of 811 persons (56).

Between-group comparisons were made for the start of attention training, subacute group (SAG) vs post-acute group (PAG) and type of intervention (APT vs ABAT), as well as combined for the start of attention training and type of intervention (SAG-APT, SAG-ABAT, PAG-APT, PAG-ABAT). One-way analysis of variance (ANOVA) and Tukey-Kramer post hoc analyses were used to compare subgroups for all outcome measures. Effect sizes (ES) (64) were calculated according to Cohen’s d (ES D) (small = 0.2, medium = 0.5, large = 0.8). Levene’s Test for Equality of Variances measured the homogeneity of variances within groups. The statistical significance level was set at *p* ˂ 0.05 2-tailed for all analyses. IBM Corp. Released 2013. IBM SPSS Statistics for Windows version 22.0 Armonk, NY: IBM Corp. was used for statistical analysis.

### Ethics

The study was approved by the Karolinska Ethics Committee (2014/1270-32) and was performed according to the principles of the Declaration of Helsinki.

## RESULTS

No differences were found between the SAG and PAG groups, nor between APT and ABAT regarding age, marital status, education, employment, or injury-related characteristics. There was a sex difference, with more men in the SAG group than the PAG group (Pearson χ^2^ 5.725, df = 1, *p* = 0.017) and more women in the APT group than the ABAT group (Pearson χ^2^ 4.944, df = 1, *p* = 0.026). The initial APT test performance was lower for PAG than for SAG (t(98) = 3.367, *p* = 0.001). No differences in the APT test were found between the APT and ABAT groups. No impacts of sex and performance on the APT test were found on analysis of co-variance.

### Health-related quality of life (EuroQol 5 dimensions)

**Fig. 1:**
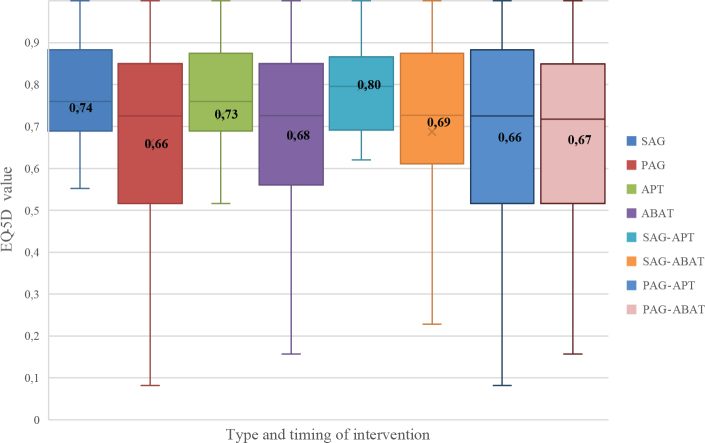
EuroQol 5 dimensions (EQ-5D) index values for timing and type of intervention. Boxplots represent the distribution between the first and third quartile within each group, with the median as centreline and inserted mean values. Swedish reference data for EQ-5D index values in mean (standard deviation; SD) for a healthy population (*n* = 25,867; aged 30–104 years) is 0.898 (0.112) for men, and 0.886 (0.116) for women (54). SAG (sub-acute group), PAG (post-acute group), APT (attention process training), ABAT (activity-based attention training).

There were no statistically significant differences in EQ-5D index values between SAG and PAG groups nor between APT and ABAT (Mann–Whitney *U* test) groups. Equal variances within groups are not assumed (F(3,96) = 3,60, *p* = 0.016) with SAG-APT showing less within-group variability (t(52) = 2.120, *p* = 0.039). The EQ-5D index values for the subgroups according to timing and type of intervention are shown in Fig. 1. SAG-APT had the highest mean HRQoL (0.80) and PAG-APT had the lowest (0.66).

**Fig. 2:**
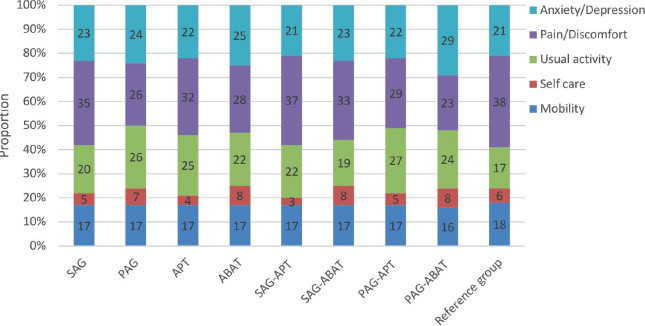
Profile of proportions of participants (%), *n* = 100, reporting problems in the 5 EuroQol 5 dimensions (EQ-5D) dimensions. Groups and subgroups of timing and type of intervention are indicated, and a reference group of healthy general population, *n* = 25,867 (54). SAG (sub-acute group), PAG (post-acute group), APT (attention process training), ABAT (activity-based attention training).

Examination of differences regarding type and timing for the individual variables in EQ-5D indicated that participants from SAG reported fewer problems doing usual activities, χ^2^ (1, N = 100) = 6.578, *p* = 0.016). Eighty-eight percent of the participants did not have problems with self-care, and 60% of the group reported an HRQoL corresponding to a healthy population. Proportions of participants reporting problems in the 5 EQ-5D dimensions are shown in Fig. 2.

### Occupational Gaps Questionnaire

The number of OGs varied between the 4 subgroups (Table III). In the SAG group, there were significantly more participants with no OGs (t(98) = 2.008, *p* = 0.047) than in the other 3 subgroups, including reporting fewer OGs (t(98) = –2.531, *p* = 0.031) than the median of an age-corrected healthy population. Conversely, PAG (t(98) = –2.252, *p* = 0.027) and PAG-APT groups reported significantly more OGs (F(3,98) = –2.054, *p* = 0.045) than other subgroups. There was no significant difference in OGs reported by the APT and ABAT groups.

**Table III:** Numbers and percentages of participants (*n* = 100), according to their number of occupational gaps (OG) in relation to median number of OGs in an age-matched healthy reference group (86)

Occupational gaps (OGs)*	Timing of intervention		Type of intervention		Type and timing of intervention			
	SAG *n* (%)	PAG *n* (%)	APT *n* (%)	ABAT *n* (%)	SAG-APT *n* (%)	SAG-ABAT *n* (%)	PAG-APT *n* (%)	PAG-ABAT *n* (%)
Participants with no gaps	7 (13)	4 (9)	8 (15)	3 (7)	5 (19)	2 (8)	3 (11)	1 (5)
Participants with 1 OG up to the median number of gaps	23 (43)	11 (23)	22 (41)	15 (32)	12 (44)	12 (42)	7 (26)	4 (20)
Participants with greater than the median number of gaps	23 (43)	32 (68)	24 (44)	28 (61)	10 (37)	13 (50)	17 (63)	15 (75)

* Median number of OGs in the reference population were age 20–29 years = 5 OGs, age 30–49 years = 4 OGs, age 50–64 years = 2 OGs, age > 65 years = 1 OG. For the reference population as a total, median number of OGs = 3.

SAG: sub-acute group; PAG: post-acute group; APT: attention process training; ABAT: activity-based attention training.

**Fig. 3:**
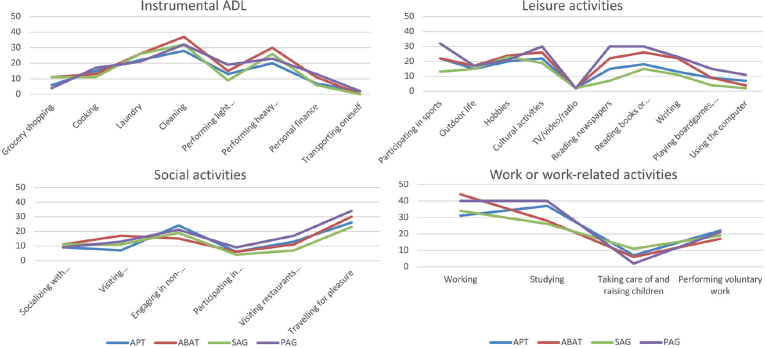
Frequency of reported occupational gaps (limitations in activity) in the Occupational Gaps Questionnaire in the different domains of activities/participations for type and timing of intervention, respectively. Distribution is presented in percentage. Low percentage signifies a better functional level. SAG: sub-acute group; PAG: post-acute group; APT: attention process training; ABAT: activity-based attention training.

The same frequency pattern of reported OGs (Fig. 3) was found in Instrumental ADL, Leisure activities, Social Activities, and Work- or work-related activities between SAG and PAG groups. The same was true for APT and ABAT groups. The highest frequency of OGs concerned cleaning, performing heavy household tasks, cultural activities and reading. Fewer problems were reported in grocery shopping, transport, meeting relatives and friends and taking care of and raising children. The SAG group reported fewer OGs in the domain of Leisure activities: “Participating in sports” (t(1) = 5.08, *p* = 0.024) and “Reading a newspaper” (t(1) = 3.348, *p* = 0.004) as compared to other subgroups. No other differences were found between study arms. The distribution of OGs in order of prevalence for all participants is shown in Appendix S1.

### Self-reported employment conditions

The SAG group (Pearson χ^2^ = 11.926, df = 1, *p* = 0.001) reported a higher prevalence (47%) of successful RTW, defined as gainful employment at ≥ 75%, compared with the PAG group (22 %). The SAG-ABAT group reported successful RTW (Pearson χ^2^ = 13.354, df = 3, *p* = 0.003), compared with subgroups. There were no differences in RTW between APT and ABAT.

Employment conditions for the subgroups are shown in Table IV. The proportion of participants reporting working at the same work having the same or adapted work tasks varied greatly between subgroups (35–74%), with more participants in SAG, APT and SAG-APT groups, respectively, reporting the same or adapted work (63–74%). Fourteen participants reported having no gainful employment, and 17 had changed jobs due to health reasons.

**Table IV:** Self-reported employment conditions at follow-up for timing and type of intervention

Variable	Timing of intervention		Type of intervention		Type and timing of intervention			
	SAG *n* = 53 *n* (%)	PAG *n* = 47 *n* (%)	APT *n* = 54 *n* (%)	ABAT *n* = 46 *n* (%)	SAG-APT *n* = 27 *n* (%)	SAG-ABAT *n* = 26 *n* (%)	PAG-APT *n* = 27 *n* (%)	PAG-ABAT *n* = 20 *n* (%)
*Actual work situation*								
Employed or self-employed	32 (60)	21 (45)	28 (52)	25 (54)	16 (60)	16 (62)	12 (44)	9 (45)
Employee 75–100%^a^	25 (47)	7 (22)	16 (30)	16 (35)	11 (41)	14 (54)	5 (19)	2 (10)
Not in gainful employment	5 (9)	9 (19)	8 (15)	6 (13)	2 (7)	3 (12)	6 (22)	3 (15)
Work retraining	2 (4)	2 (4)	5 (9)	5 (11)	2 (7)	6 (23)	4 (15)	5 (20)
Working at the same work (same work tasks)	21 (40)	15 (32)	23 (43)	13 (28)	13 (48)	8 (31)	10 (37)	5 (20)
Working at the same work (adapted work tasks)^b^	14 (26)	6 (13)	11 (20)	9 (20)	7 (26)	7 (27)	4 (15)	2 (10)
Changed job due to health reasons	7 (13)	10 (21)	6 (11)	11 (24)	1 (4)	6 (23)	5 (19)	5 (20)
*Sick benefit or activity compensation*								
No sickness benefit or activity compensation	44 (83)	35 (74)	47 (87)	32 (70)	24 (89)	23 (88)	20 (74)	12 (60)
Sickness benefit or activity compensation 25–50%	1 (2)	5 (12)	4 (7)	2 (8)	1 (4)	2 (8)	0 (0)	5 (20)
Sickness benefit or activity compensation ≥ 75%	7 (15)	6 (13)	3 (5)	10 (22)	2 (7)	1 (4)	5 (26)	5 (20)

a Participants (*n* = 5) working at 75% also receive activity compensation at 25%.

b Participants on work retraining (*n* = 17) are included in this group.

The participants could select more than 1 option in the questionnaire.

SAG: sub-acute group; PAG: post-acute group; APT: attention process training; ABAT: activity-based attention training.

Forty-six percent of all participants self-reported receiving sickness benefits or sickness compensation to some degree, 70% of whom (*n* = 33) received full payment due to disability. In Sweden, if a person has an RTW less than full-time, they can receive sickness compensation for the remainder up to 100%. No significant difference existed between groups and subgroups for the self-reported degree of sick leave, sickness allowance or sickness compensation.

### Work Ability Index

The mean WAI Total score for the different subgroups varied between 29 and 33 points, corresponding to moderate work ability, implying a “need to improve” work ability.

SAG reported better work ability (WAI Total Score; independent sample Mann–Whitney *U* test, *p* = 0.00) with a strong ES D (Cohen’s d = –0.92) and higher self-rated work ability (WAS = t(92)3,338, *p* = 0.001) (Table V) than PAG.

**Table V:** Performance on Work Ability Index, showing mean (standard deviation; SD) and median for timing and type of intervention for all participants, for Total Score and for sub-dimensions separately

	Reference* *n* = 1,786 Mean	Timing of intervention		Type of intervention	
		SAG (*n* = 53) Mean (SD)/md	PAG (*n* = 47) Mean (SD)/md	APT (*n* = 54) Mean (SD)/md	ABAT (*n* = 46) Mean (SD)/md
WAI Total Score (*n* = 74)^1^	41.53	36 (8)/38	29 (9)/29	33 (9)/33	33 (9)/34
*Individual resources*					
a. Current WA compared with estimated best (WAS)	8.25	6 (3)/8	4 (3)/5	5 (3)/6	5 (3)/6
b^1^. WA in relation to physical demands (current work)	4.29	4 (1)/4	4 (1)/4	4 (1)/4	4 (1)/4
b^2^. WA in relation to mental demands (current work)	4.23	4 (1)/4	3 (1)/4	4 (1)/4	4 (1)/4
f. Own prognosis of WA (2 years)	6.64	6 (2)/7	5 (2)/7	5 (2)/7	6 (2)/7
g. Enjoying daily activities (mental resources)	2,9	3 (1)/3	3 (1)/3	3 (1)/3	3 (1)/3
WAI Individual Health Factor^2^					
c. Number of medical diagnoses	5.82	4 (1)/4	3 (1)/3	4 (1)/4	4 (1)/4
d. Estimated WA impairment due to diseases	5.24	3 (1)/3	2 (1)/2	3 (1)/3	2 (1)/3
e. Sick leave (last 12 months)	4.15	3 (1)/3	3 (1)/2	3 (2)/2	3 (2)/2

1 Total score for the WAI dimensions a, b^1^, b^2^, f, g;

2 Total score for WAI dimensions c, d, e;

* Values of reference from a healthy general population (58).

SAG: sub-acute group; PAG: post-acute group; APT: attention process training; ABAT: activity-based attention training; SD: standard deviation; md: median.

There were no differences in the WAI Total Score or the WAS, depending on the interventions APT or ABAT only. However, SAG-APT reported better outcome on the WAI Total Score (F(3,76) = 4,755, *p* = 0.004) and the WAS (F(3,90) = 3,802, *p* = 0.013).

The total WAI score (Table VI) grouped the participants into 4 classes, which showed that SAG (62%) self-assessed their work ability to be “good” or “excellent” to a higher degree than PAG (19%) (Fisher’s χ^2^ test, *p* = 0.001; post hoc independent-sample Mann–Whitney *U* test, *p* = 0.000). No statistical differences were found between the APT and ABAT groups. When comparing the subgroups, the SAG-APT group reported a higher self-assessed level of work ability (t(3) = 4.996, *p* = 0.003).

**Table VI:** Distribution of participants sorted into 4 levels of working ability (poor, moderate, good, excellent) based on the total score according to the Work ability Index (WAI) Manual and in relation to timing and type of intervention respectively

WAI, %	Levels of working ability	Timing of intervention		Type of intervention		Type and timing of intervention			
		SAG *n* = 42 %	PAG *n* = 32 %	APT *n* = 37 %	ABAT *n* = 37 %	SAG-APT *n* = 21 %	PAG-APT *n* = 16 %	SAG-ABAT *n* = 21 %	PAG-ABAT *n* = 16 %
Poor work ability	(7–27)	14	41	27	24	14	44	14	38
Moderate work ability	(28–36)	24	41	30	32	19	44	28	38
Good work ability	(37–43)	48	13	30	35	48	6	48	19
Excellent work ability	(44–49)	14	6	14	8	19	6	10	6

SAG: sub-acute group; PAG: post-acute group; APT: attention process training; ABAT: activity-based attention training.

### Experienced limitations in work performance

Participants in the different groups reported cognitive and behavioural problems (Table VII), the most frequent issues concerned being more easily disrupted at work, more easily getting tired, experiencing work tasks taking longer, and concentration problems at work. The PAG group reported more problems with work tasks taking longer time than expected (Pearson χ^2^ = 10.361, df = 2, *p* = 0.006). Participants receiving the APT reported fewer problems due to forgetting things at work (Pearson χ^2^ = 6.428, df = 2, *p* = 0.040) and keeping the workplace tidy (Pearson χ^2^ = 6.887, df = 2, *p* = 0.032). The SAG-APT group (t(3) = 15.910, *p* = 0.013) reported fewer problems keeping their workplace tidy.

**Table VII:** Experienced limitations in work performance at follow-up for timing and type of intervention. Experienced limitations are presented in order of prevalence for all participants

Single questions related to experienced limitations at work, *n* (%)	Timing of intervention		Type of intervention		Timing and type of intervention			
	SAG *n* = 45 *n* (%)	PAG *n* = 33 *n* (%)	APT *n* = 40 *n* (%)	ABAT *n* = 38 *n* (%)	SAG-APT *n* = 22 *n* (%)	PAG-APT *n* = 18 *n* (%)	SAG-ABAT *n* = 23 *n* (%)	PAG-ABAT *n* = 15 *n* (%)
I easily get disturbed at work	32 (71)	30 (91)	31 (78)	31 (81)	15 (68)	16 (89)	17 (74)	14 (93)
I easily get tired	31 (69)	28 (85)	31 (78)	28 (74)	15 (68)	16 (89)	16 (70)	12 (80)
Work tasks take longer time	30 (67)	26 (79)	30 (75)	26 (68)	16 (73)	14 (82)	14 (61)	12 (80)
It is difficult to concentrate on work tasks	25 (56)	22 (67)	23 (58)	24 (63)	12 (55)	12 (67)	14 (61)	10 (67)
I make careless mistakes	22 (50)	20 (61)	23 (58)	19 (51)	10 (45)	13 (72)	12 (52)	7 (47)
I forget to do things at work	18 (40)	19 (58)	16 (40)	21 (55)	7 (32)	9 (50)	11 (48)	10 (67)
I find it difficult initiating/ structuring my work	18 (40)	18 (54)	14 (35)	22 (58)	6 (27)	8 (44)	12 (52)	10 (67)
Relationships with co-workers have changed	16 (36)	15 (48)	16 (42)	15 (41)	8 (36)	8 (44)	8 (35)	7 (47)
I misunderstand instructions	13 (29)	12 (36)	11 (28)	14 (37)	5 (23)	6 (33)	8 (35)	6 (40)
I have difficulties in keeping up with time	14 (31)	11 (32)	16 (40)	9 (24)	9 (41)	7 (39)	5 (22)	5 (27)
I have difficulties keeping my workplace tidy	10 (22)	14 (42)	7 (18)	17 (45)	1 (5)	6 (18)	9 (39)	6 (40)
Pain affects my work performance	11 (24)	10 (30)	13 (33)	8 (21)	6 (27)	7 (39)	5 (22)	3 (20)
I have problems performing my work tasks	5 (11)	7 (21)	5 (13)	7 (20)	2 (9)	3 (17)	3 (14)	4 (27)

Results are presented in number (%) of answers reported as “yes” and “sometimes”. SAG: sub-acute group; PAG: post-acute group; APT: attention process training; ABAT: activity-based attention training.

All study participants experienced support from their co-workers, and 92% reported having supportive managers.

## DISCUSSION

An important finding was that participants starting attention training within 4 months post-ABI reported significantly higher outcomes on HRQoL and work participation and fewer activity limitations. However, only minor differences were found for a specific intervention programme, favouring the advantage of APT within 4 months post-injury.

The results strengthen earlier findings concerning the importance of cognitive rehabilitation within the first year after ABI for functional outcome (27, 28, 32), psychological well-being (30), and vocational functioning (29, 65). Our data strongly support cognitive rehabilitation within the first 3–4 months after ABI for patients with attention impairment.

Furthermore, the subacute group perceived fewer restrictions in everyday occupations (43% vs 68%). No study was found in the literature review for the current study comparing a subacute vs a post-acute intervention group on the results of limitations in daily occupations. The time intervals between rehabilitation and follow-up in existing studies, deviate too much from our study to enable comparisons (56, 66, 67, 68).

The subacute and post-acute groups followed the same pattern when reporting restrictions in different daily occupations (Fig. 3). However, our subacute group reported fewer restrictions for “hobbies” (15%) in contrast to our post-acute group (32 %) and to Bergstrom’s study (32%) (68). The results showed that sub-acute cognitive rehabilitation resulted in higher self-rated work ability (e.g., index value), especially for those receiving APT. Work ability seems linked to functional impairments, attitudes towards disability, and motivation to work (69) and depends on the person’s insight into injury-related problems that might influence work performance (70). In an earlier study on the management of attention (14) we found that increased self-awareness, paired with coping strategies at an early stage, potentially mitigates expedience in performance despite disability and perceived limitations in activity, which could account for the findings of higher WAI for the APT group. However, we can only hypothesize why the impact seems greater with sub-acute rehabilitation.

The comparisons showed a higher prevalence of successful RTW in patients receiving sub-acute intervention than post-acute intervention. RTW is essential for QoL (34) and is a realistic goal for many working-age patients with ABI. Thus, RTW rates are of interest in rehabilitation research, even if the interpretation beyond the usual individual functioning level needs to be careful since societal factors influence it to varying degrees. When comparing RTW rates in studies from different countries, comparisons need to respect the differences in each country. The RTW rate of 47% for gainful employment ≥ 75% is well within the range of earlier reports (43, 71–73). No study has been found comparing the sub- vs post-acute attention-training long-term outcome on RTW. Regarding RTW, the current study results do not support selecting one training method above the other. Cognitive function (38, 74–76), independence in ADL, together with pre-injury factors, such as education and type of job, and perceived work ability (76) are identified as predictive factors (75, 77) for successful RTW. Although attention dysfunction has proven to have a significant impact on RTW within 18 months post-onset (38), studies identifying the potential impact of specific cognitive disability on RTW (78) or outcome of RTW post rehabilitation (74) are scarce. No study has been found comparing the 2 attention-training methods’ effects on long-term RTW.

In the current study, there were only minor statistical differences between the 2 attention interventions using measures of activity and participation, which was unexpected. Previously, our research group reported the advantage of APT on a neuropsychological outcome measure, Paced Auditory Serial Addition Test (21), using a statistical process method (49). On the level of activity and participation, using a standardized office-work task and evaluated according to the Assessment of Work Performance measure (AWP) (79, 80), significant differences were found, favouring APT post-intervention and at a 3-month follow-up for some process skills (Mental Energy, Knowledge, Temporal Organization, Adaptation and Physical Energy) (20). AWP assesses an individual’s observable skills during work performance, i.e., how efficiently and appropriately a client performs a work activity. These skills are subserved by executive and attention functions trained in the programme, suggesting some transfer effects of the training from body to activity levels. For other outcome measures, such as the WAI, a significant improvement was observed after intensive rehabilitation of attention. However, there were no differences between the 2 training methods, as observed in the current study for WAI.

It should be noted that the minor differences observed in the current study supporting APT, and particularly in the subacute group (SAG-APT has the highest QoL, highest successful RTW, better outcome on WAI Total Score and fewer problems in organizing their workplace). In addition, APT patients reported fewer memory problems at work. The only discrepant result was significantly more OGs for PAG-APT.

The results of the current study can be interpreted in several contexts: (*i*) differences between process-based and activity-based attention training might disappear gradually. As time passes, practical behavioural elements, strategies and skills acquired in activity-based training get individually incorporated into everyday behaviour according to situations, needs and preferences. This slow process might smooth out early differences in the effects of training methods in the long-term. Patients utilize rehabilitation programmes differently due to their strengths and weaknesses, leading to a gradual absorption of the newly learned behavioural elements or performance skills in daily life. This process makes the effects of underlying cognitive training in one area (attention) challenging to discern. In our earlier interview study with participants receiving a multidisciplinary rehabilitation programme and APT (14), we provided examples of how patients apply APT training after discharge from rehabilitation. (*ii*) Conceptual differences in measures may be another reason for the different results. Earlier studies found low to moderate correlations between measures for cognitive tests (body-function level) and the Assessment of Motor and Process Skills (AMPS) activity level (81). As discussed previously (80), some outcome measures on activity level, such as QoL and work ability, are global measures primarily assessing the impact of a disease without referring to specific behaviours. The effects of a directed cognitive intervention might not be easily detected in such a context.

Another category of outcome measures on activity level is performance skills, defined as small observable units of behaviour used to perform a specific task (82) and offer higher conceptual proximity to variables in a neuropsychological test situation. However, these behavioural units, such as time management and structuring one’s workstation, require attentional support to varying extents. Improvements in attention, a cognitive function, are thus differentially reflected in activity changes as a function of task requirements and individual qualifications. Furthermore, these measurement instruments, such as AMPS and AWP, allow the selection of different tasks or activities. Hence, differences in types of activities make group comparisons more problematic.

An additional group of activity-based measures are functionally based standardized performance-based instruments directly relevant to clinically meaningful outcomes (6). Our standardized office-task targeting skills in attention, organization, and processing skills is an example of this approach (80). The task follows a formalized procedure with standardized scoring according to predefined criteria and values for a healthy comparison group.

Due to earlier criticism regarding the lack of evidence in the generalization of cognitive training to real-life activities (83) and to answer the calling of earlier reviews (84, 85) we have selected outcome measures on the level of activity and participation in the current study. Our varying results suggest a need to distinguish between different subcategories and choose measures closely related to the target of rehabilitation, i.e., performance-based measurements, as recommended (6).

When relating the current study results to healthy reference groups for each measure, the results indicate that the subacute group rates their QoL at the same level as the healthy population and has few restrictions in daily occupations. However, participants in the current study rated their work ability (mean WAI total score) as “moderate” work ability (need to improve) in contrast to a healthy reference group ranking theirs as “good” work ability (59). The lower ratings may be interpreted as that, after 2 years, the patients still needed measures to improve their work ability, especially the 26% of the participants who experienced poor work ability.

### Study limitations

First, due to the recommendation for homogeneous groups (50) the current study participants showed a restricted range of clinical symptoms, excluding patients with comorbidities, including higher severity of injury and aphasia. The current results are based on patients with mild to moderate deficits post-ABI, so the results do not apply to patients with more severe injuries and complexity in cognitive impairment.

The dropouts from the current study had lower results on logical thinking at the time of intervention, which we consider having minimal influence on the conclusions.

These results are based on self-report data regarding the working situation and social support. A third limitation thus concerns the choice of measures, as self-reports are not always reliable, due to the complexity of these measures. Independent data sources, such as registered data from insurance agencies, are recommended to give as correct data as possible on sick leave days and sickness compensation extent and periods. However, no such resources were available. A strength of the current study was the telephone-based follow-up for completing the reports, circumventing the most common confounders in self-reports, i.e., missing data and the influence of next-of-kin.

### Conclusion

This study followed up working-age patients on daily activities, RTW and work ability 2 years after they had undergone intensive attention training for moderate attention impairment post-ABI. The results emphasize the importance of subacute (≤ 4 months) over post-acute (4–12 months) start of rehabilitation. When comparing attention training interventions, no differences could be distinguished except for a few minor advantages that could be demonstrated for APT in the group who underwent subacute rehabilitation. Valid measurements of how impaired functions influence activities and participation in the long term are complex and require further research.

### Clinical implications

The results of this study provide information concerning long-term outcomes of subacute intensive attention training regarding QoL and aspects of working capacity for patients with ABI of working age. These results contribute to the accumulating body of knowledge concerning the importance of early rehabilitation. According to current clinical practice, patients are mobilized and activated during the acute inpatient and rehabilitation phases and then, after housing adaptation, often discharged to home-based rehabilitation. However, the resources of the teams providing home-based rehabilitation are not sufficient to meet the requirements of intensive attention training. These results indicate the need for providing more patients with shorter periods of intensive sub-acute outpatient rehabilitation.

Differences between training methods seemed to attenuate over time. However, the clinician should note that both APT and ABAT were accompanied by extensive metacognitive training and tasks focusing on attention-demanding activities, as recommended by several guidelines (6, 7, 45).

## Supplementary Material

REHABILITATION WITH INTENSIVE ATTENTION TRAINING EARLY AFTER ACQUIRED BRAIN INJURY PROMOTES BETTER LONG-TERM STATUS ON HEALTH-RELATED QUALITY OF LIFE, DAILY ACTIVITIES, WORK ABILITY AND RETURN TO WORK

## References

[1] Ponsford J, Bayley M, Wiseman-Hakes C, Togher L, Velikonja D, McIntyre A, et al. INCOG recommendations for management of cognition following traumatic brain injury, part II: attention and information processing speed. J Head Trauma Rehabil 2014; 29: 321–337. 24984095 10.1097/HTR.0000000000000072

[2] Barker-Collo S, Feigin V, Lawes C, Senior H, Parag V. Natural history of attention deficits and their influence on functional recovery from acute stages to 6 months after stroke. Neuroepidemiol 2010; 35: 255–262. 10.1159/000319894 20881428

[3] Söderback I, Ekholm J, Caneman G. Impairment/function and disability/activity 3 years after cerebrovascular incident or brain trauma: a rehabilitation and occupational therapy view. Int Disabil Stud 1991; 13: 67–73. 1837792 10.3109/03790799109166687

[4] Virk S, Williams T, Brunsdon R, Suh F, Morrow A. Cognitive remediation of attention deficits following acquired brain injury: a systematic review and meta-analysis. NeuroRehabilitation 2015; 36: 367–377. 26409340 10.3233/NRE-151225

[5] Sohlberg MM, CA Cognitive Rehabilitation: an integrative neuropsychological approach. New York: The Guilford Press; 2001.

[6] Cicerone KD, Dams-O’Connor K, Eberle R, Ganci K, Langenbahn DM, Shapiro-Rosenbaum A, et al. The ACRM cognitive rehabilitation manual & textbook. translating evidence-based recommendations into practice. Second edition. Reston, VA, USA ACRM Publishing; 2022.

[7] Ponsford J, Velikonja D, Janzen S, Harnett A, McIntyre A, Wiseman-Hakes C, et al. INCOG 2.0 guidelines for cognitive rehabilitation following traumatic brain injury, Part II: attention and information processing speed. J Head Trauma Rehabil 2023; 38: 38–51. 36594858 10.1097/HTR.0000000000000839

[8] Sohlberg MM, Mateer CA. Effectiveness of an attention-training program. J Clin Exp Neuropsychol 1987; 9: 117–130. 3558744 10.1080/01688638708405352

[9] Barker-Collo SL, Feigin VL, Lawes CM, Parag V, Senior H, Rodgers A. Reducing attention deficits after stroke using attention process training: a randomized controlled trial. Stroke 2009; 40: 3292–3298. 10.1161/STROKEAHA.109.558239 19628801

[10] Cicerone KD, Goldin Y, Ganci K, Rosenbaum A, Wethe JV, Langenbahn DM, et al. Evidence-based cognitive rehabilitation: systematic review of the literature from 2009 through 2014. Arch Phys Med Rehabil 2019; 100: 1515–1533. 30926291 10.1016/j.apmr.2019.02.011

[11] Cicerone KD, Langenbahn DM, Braden C, Malec JF, Kalmar K, Fraas M, et al. Evidence-based cognitive rehabilitation: updated review of the literature from 2003 through 2008. Arch Phys Med Rehabil 2011; 92: 519–530. 21440699 10.1016/j.apmr.2010.11.015

[12] Engle JA, Kerns KA. Reinforcement learning in children with FASD. J Popul Ther Clin Pharmacol 2011; 18: e17–27. 21289375

[13] Cicerone KD, Dahlberg C, Kalmar K, Langenbahn DM, Malec JF, Bergquist TF, et al. Evidence-based cognitive rehabilitation: recommendations for clinical practice. Arch Phys Med Rehabil 2000; 81: 1596–1615. 11128897 10.1053/apmr.2000.19240

[14] Markovic G, Bartfai A, Ekholm J, Nilsson C, Schult ML, Löfgren M. Daily management of attention dysfunction two-four years after brain injury and early cognitive rehabilitation with attention process training: a qualitative study. Neuropsychol Rehabil 2020; 30: 523–544. 29947254 10.1080/09602011.2018.1482770

[15] Pantoni L, Poggesi A, Diciotti S, Valenti R, Orsolini S, Della Rocca E, et al. Effect of attention training in mild cognitive impairment patients with subcortical vascular changes: The RehAtt Study. J Alzheimers Dis 2017; 60: 615–624. 28869475 10.3233/JAD-170428 PMC5611829

[16] Govender P, Kalra L. Benefits of occupational therapy in stroke rehabilitation. Expert Rev Neurother 2007; 7: 1013–1019. 17678496 10.1586/14737175.7.8.1013

[17] Park HY, Maitra K, Martinez KM. The effect of occupation-based cognitive rehabilitation for traumatic brain injury: a meta-analysis of randomized controlled trials. Occup Ther Int 2015; 22: 104–116. 25808426 10.1002/oti.1389

[18] Bartfai A, Markovic G, Sargenius Landahl K, Schult ML. The protocol and design of a randomised controlled study on training of attention within the first year after acquired brain injury. BMC Neurol 2014; 14: 102. 24885585 10.1186/1471-2377-14-102 PMC4018266

[19] Sargénius Landahl K. Evaluation of Attention training after acquired brain injury – an occupational perspective [rehabilitation medicine]. ProQuest Dissertations Publishing: Karolinska Institutet; 2021, Stockholm, Sweden.

[20] Sargénius Landahl K, Schult ML, Borg K, Bartfai A. Comparison of attention process training and activity-based attention training after acquired brain injury: a randomized controlled study. J Rehabil Med 2021; 53: jrm00235. 34554264 10.2340/16501977-2875 PMC8638745

[21] Markovic G, Schult ML, Elg M, Bartfai A. Beneficial effects of early attention process training after acquired brain injury: a randomized controlled trial. J Rehabil Med 2020; 52: jrm00011. 10.2340/16501977-2628 31742648

[22] Bartfai A, Elg M, Schult ML, Markovic G. Predicting outcome for early attention training after acquired brain injury. Front Hum Neurosci 2022; 16: 767276. 35664351 10.3389/fnhum.2022.767276 PMC9159897

[23] McCrea MA, Giacino JT, Barber J, Temkin NR, Nelson LD, Levin HS, et al. Functional outcomes over the first year after moderate to severe traumatic brain injury in the prospective, longitudinal TRACK-TBI study. JAMA Neurol 2021; 78: 982–992. 34228047 10.1001/jamaneurol.2021.2043 PMC8261688

[24] Gilmore N, Katz DI, Kiran S. Acquired brain injury in adults: a review of pathophysiology, recovery, and rehabilitation. Perspect ASHA Spec Interest Groups 2021; 6: 714–727. 34746412 10.1044/2021_persp-21-00013 PMC8570578

[25] Rabadi MH, Rabadi FM, Edelstein L, Peterson M. Cognitively impaired stroke patients do benefit from admission to an acute rehabilitation unit. Arch Phys Med Rehabil 2008; 89: 441–448. 18295621 10.1016/j.apmr.2007.11.014

[26] Fan MC, Li SF, Sun P, Bai GT, Wang N, Han C, et al. Early intensive rehabilitation for patients with traumatic brain injury: a prospective pilot trial. World Neurosurg 2020; 137: e183–e188. 32001397 10.1016/j.wneu.2020.01.113

[27] Hayden ME, Plenger P, Bison K, Kowalske K, Masel B, Qualls D. Treatment effect versus pretreatment recovery in persons with traumatic brain injury: a study regarding the effectiveness of postacute rehabilitation. PM & R 2013; 5: 319–327; quiz 27. 23375632 10.1016/j.pmrj.2012.12.005

[28] León-Carrión J, Machuca-Murga F, Solís-Marcos I, León-Domínguez U, Domínguez-Morales Mdel R. The sooner patients begin neurorehabilitation, the better their functional outcome. Brain Inj 2013; 27: 1119–1123. 23895589 10.3109/02699052.2013.804204

[29] Reid-Arndt SA, Schopp L, Brenneke L, Johnstone B, Poole AD. Evaluation of the traumatic brain injury early referral programme in Missouri. Brain Inj 2007; 21: 1295–1302. 17963093 10.1080/02699050701721802

[30] Saux G, Demey I, Rojas G, Feldberg C. Cognitive rehabilitation therapy after acquired brain injury in Argentina: psychosocial outcomes in connection with the time elapsed before treatment initiation. Brain Inj 2014; 28: 1447–1454. 24865389 10.3109/02699052.2014.919528

[31] Schultz R, Tate RL, Perdices M. Neuropsychological recovery during the first 12 months after severe traumatic brain injury: a longitudinal study with monthly assessments. Neuropsychol Rehabil 2022;32: 1291–1323. 33685355 10.1080/09602011.2021.1882507

[32] Zarshenas S, Colantonio A, Horn SD, Jaglal S, Cullen N. Cognitive and motor recovery and predictors of long-term outcome in patients with traumatic brain injury. Arch Phys Med Rehabil 2019; 100: 1274–1282. 30605639 10.1016/j.apmr.2018.11.023

[33] Lo Buono V, Corallo F, Bramanti P, Marino S. Coping strategies and health-related quality of life after stroke. J Health Psychol 2017; 22: 16–28. 26220458 10.1177/1359105315595117

[34] Westerlind E, Persson HC, Törnbom K, Sunnerhagen KS. Return to work predicts perceived participation and autonomy by individuals with stroke. Disabil Rehabil 2020; 42: 3673–3678. 31068023 10.1080/09638288.2019.1608324

[35] Vestling M, Tufvesson B, Iwarsson S. Indicators for return to work after stroke and the importance of work for subjective well-being and life satisfaction. J Rehabil Med 2003; 35: 127–131. 12809195 10.1080/16501970310010475

[36] Donker-Cools BH, Daams JG, Wind H, Frings-Dresen MH. Effective return-to-work interventions after acquired brain injury: a systematic review. Brain Inj 2016; 30: 113–131. 26645137 10.3109/02699052.2015.1090014

[37] Gorgoraptis N, Zaw-Linn J, Feeney C, Tenorio-Jimenez C, Niemi M, Malik A, et al. Cognitive impairment and health-related quality of life following traumatic brain injury. NeuroRehabil 2019; 44: 321–331. 10.3233/NRE-182618 31177238

[38] Tanaka H, Toyonaga T, Hashimoto H. Functional and occupational characteristics predictive of a return to work within 18 months after stroke in Japan: implications for rehabilitation. Int Arch Occup Environ Health 2014; 87: 445–453. 23677520 10.1007/s00420-013-0883-8 PMC3996276

[39] Dawson DR, Levine B, Schwartz ML, Stuss DT. Acute predictors of real-world outcomes following traumatic brain injury: a prospective study. Brain Inj 2004; 18: 221–238. 14726283 10.1080/02699050310001617406

[40] Mateer CA, Sira CS. Cognitive and emotional consequences of TBI: intervention strategies for vocational rehabilitation. NeuroRehabil 2006; 21: 315–326. 17361048

[41] Vilkki JS, Juvela S, Siironen J, Ilvonen T, Varis J, Porras M. Relationship of local infarctions to cognitive and psychosocial impairments after aneurysmal subarachnoid hemorrhage. Neurosurg 2004; 55: 790–802; discussion –3. 10.1227/01.neu.0000137629.17424.6d 15458587

[42] Willers C, Westerlind E, Borgström F, von Euler M, Sunnerhagen KS. Health insurance utilisation after ischaemic stroke in Sweden: a retrospective cohort study in a system of universal healthcare and social insurance. BMJ Open 2021; 11: e043826. 10.1136/bmjopen-2020-043826 PMC7993163 33762236

[43] Westerlind E, Persson HC, Eriksson M, Norrving B, Sunnerhagen KS. Return to work after stroke: a Swedish nationwide registry-based study. Acta Neurol Scand 2020; 141: 56–64. 31659744 10.1111/ane.13180 PMC6916554

[44] Tate RL, Simpson GK, McRae P. Return to work after trauamtic brain injury: a literature review. In: Escorpizo R, Brage S, Homa D, Stucki G, editors. Handbook of vocational rehabilitation and disability evaluation: application and implementation of the ICF 2015th edn. Springer International Publishing, Switzerland 2015, p. 275.

[45] Loetscher T, Potter KJ, Wong D, das Nair R. Cognitive rehabilitation for attention deficits following stroke. Cochrane Database Syst Rev 2019; 2019: CD002842. 31706263 10.1002/14651858.CD002842.pub3 PMC6953353

[46] Rogers JM, Foord R, Stolwyk RJ, Wong D, Wilson PH. General and domain-specific effectiveness of cognitive remediation after stroke: systematic literature review and meta-analysis. Neuropsychol Rev 2018; 28: 285–309. 30006801 10.1007/s11065-018-9378-4

[47] Grönholm-Nyman P, Soveri A, Rinne JO, Ek E, Nyholm A, Stigsdotter Neely A, et al. Limited effects of set shifting training in healthy older adults. Front Aging Neurosci 2017; 9: 69. 28386226 10.3389/fnagi.2017.00069 PMC5362725

[48] Zelinski EM. Far transfer in cognitive training of older adults. Restor Neurol Neurosci 2009; 27: 455–471. 19847070 10.3233/RNN-2009-0495 PMC4169295

[49] Markovic G, Schult ML, Bartfai A, Elg M. Statistical process control: a feasibility study of the application of time-series measurement in early neurorehabilitation after acquired brain injury. J Rehabil Med 2017; 49: 128–135. 27904913 10.2340/16501977-2172

[50] Markovic G, Schult ML, Bartfai A. The effect of sampling bias on generalizability in intervention trials after brain injury. Brain Inj 2017; 31: 9–15. 27819515 10.1080/02699052.2016.1206213

[51] Wiseman-Hakes C, MacDonald S, Keightley M. Perspectives on evidence based practice in ABI rehabilitation. “Relevant Research”: who decides? NeuroRehabil 2010; 26: 355–368. 10.3233/NRE-2010-0573 20555159

[52] Balestroni G, Bertolotti G. [EuroQol-5D (EQ-5D): an instrument for measuring quality of life]. Monaldi Arch Chest Dis 2012; 78: 155–159. 23614330 10.4081/monaldi.2012.121

[53] Dolan P. Modeling valuations for EuroQol health states. Med Care 1997; 35: 1095–1108. 9366889 10.1097/00005650-199711000-00002

[54] Teni FS, Gerdtham UG, Leidl R, Henriksson M, Åström M, Sun S, et al. Inequality and heterogeneity in health-related quality of life: findings based on a large sample of cross-sectional EQ-5D-5L data from the Swedish general population. Qual Res Res 2022; 31: 697–712. 10.1007/s11136-021-02982-3 PMC8921093 34628587

[55] Dorman PJ, Waddell F, Slattery J, Dennis M, Sandercock P. Is the EuroQol a valid measure of health-related quality of life after stroke? Stroke 1997; 28: 1876–1882. 9341688 10.1161/01.str.28.10.1876

[56] Eriksson G, Tham K, Kottorp A. A cross-diagnostic validation of an instrument measuring participation in everyday occupations: the Occupational Gaps Questionnaire (OGQ). Scand J Occup Ther 2013; 20: 152–160. 23216376 10.3109/11038128.2012.749944

[57] Tuomi K, Ilmarinen J, Jahkola A, Katajarinne L, Tulkki A. Work Ability Index. Finnish Institute of Occupational Health, Helsinki, Finland; 1998.

[58] Lundin A, Leijon O, Vaez M, Hallgren M, Torgén M. Predictive validity of the Work Ability Index and its individual items in the general population. Scand J Public Health 2017; 45: 350–356. 28385066 10.1177/1403494817702759

[59] Pranjic N, Gonzales JMG, Cvejanov-Kezunović L. Perceived work ability index of public service employees in relation to ageing and gender: a comparison in three European countries. Zdr Varst 2019; 58: 179–188. 31636726 10.2478/sjph-2019-0023 PMC6778419

[60] Lundin A, Kjellberg K, Leijon O, Punnett L, Hemmingsson T. The association between self-assessed future work ability and long-term sickness absence, disability pension and unemployment in a general working population: a 7-year follow-up study. J Occup Rehabil 2016; 26: 195–203. 26319413 10.1007/s10926-015-9603-4

[61] Haskins ME, Cicerone KD, Dams-O’Connor K, Langenbahn DM, Shapiro-Rosenbaum A. Cognitive rehabilitation manual. Translating evidence-based recommendations into practice. Virginia, USA: American Congress of Rehabilitation Medicine, ACRM (BI-ISIG); 2013.

[62] Polatajko HJ, McEwen SE, Ryan JD, Baum CM. Pilot randomized controlled trial investigating cognitive strategy use to improve goal performance after stroke. Am J Occup Ther 2012; 66: 104–109. 22389945 10.5014/ajot.2012.001784

[63] McEwen S, Polatajko H, Baum C, Rios J, Cirone D, Doherty M, et al. Combined cognitive-strategy and task-specific training improve transfer to untrained activities in subacute stroke: an exploratory randomized controlled trial. Neurorehabil Neural Repair 2015; 29: 526–536. 25416738 10.1177/1545968314558602 PMC4440855

[64] Cohen J. Statistical power analysis for the behavioral sciences. Hillsdale: L. Erlbaum Associates; 1988.

[65] Tanaka H, Toyonaga T, Hashimoto H. Functional and occupational characteristics associated with very early return to work after stroke in Japan. Arch Phys Med Rehabil 2011; 92: 743–748. 21530721 10.1016/j.apmr.2010.12.009

[66] Svensson JS, Westerlind E, Persson HC, Sunnerhagen KS. Occupational gaps 5 years after stroke. Brain Behav 2019; 9: 9e01234. 10.1002/brb3.1234 PMC6422817 30784220

[67] Bergström AL, von Koch L, Andersson M, Tham K, Eriksson G. Participation in everyday life and life satisfaction in persons with stroke and their caregivers 3–6 months after onset. J Rehabil Med 2015; 47: 508–515. 25882897 10.2340/16501977-1964

[68] Bergström A, Guidetti S, Tham K, Eriksson G. Association between satisfaction and participation in everyday occupations after stroke. Scand J Occup Ther 2017; 24: 339–348. 27774829 10.1080/11038128.2016.1245782

[69] Lindgren I, Pessah-Rasmussen H, Gard G, Brogårdh C. Perceived work situation and work ability among persons who are working one year after stroke. J Rehabil Med 2022; 54: jrm00254. 34825916 10.2340/jrm.v53.918 PMC8862645

[70] Hellman T, Bergström A, Eriksson G, Hansen Falkdal A, Johansson U. Return to work after stroke: important aspects shared and contrasted by five stakeholder groups. Work 2016; 55: 901–911. 28059820 10.3233/WOR-162455

[71] Scaratti C, Leonardi M, Sattin D, Schiavolin S, Willems M, Raggi A. Work-related difficulties in patients with traumatic brain injury: a systematic review on predictors and associated factors. Disabil Rehabil 2017; 39: 847–855. 28293979 10.3109/09638288.2016.1162854

[72] Watkin C, Phillips J, Radford K. What is a ‘return to work’ following traumatic brain injury? Analysis of work outcomes 12 months post TBI. Brain Inj 2020; 34: 68–77. 31661643 10.1080/02699052.2019.1681512

[73] van Velzen JM, van Bennekom CA, Edelaar MJ, Sluiter JK, Frings-Dresen MH. How many people return to work after acquired brain injury?: a systematic review. Brain Inj 2009; 23: 473–488. 19484621 10.1080/02699050902970737

[74] Wei X-J, Liu X-f, Fong KNK. Outcomes of return-to-work after stroke rehabilitation: a systematic review. Br J Occup Ther 2016; 79: 299–308.

[75] Donker-Cools B, Wind H, Frings-Dresen MHW. Prognostic factors of return to work after traumatic or non-traumatic acquired brain injury. Disabil Rehabil 2016; 38: 733–741. 26138021 10.3109/09638288.2015.1061608

[76] Saar K, Tolvanen A, Poutiainen E, Aro T. Returning to work after stroke: associations with cognitive performance, motivation, perceived working ability and barriers. J Rehabil Med 2023; 55: jrm00365. 36622215 10.2340/jrm.v55.2576 PMC9847477

[77] Edwards JD, Kapoor A, Linkewich E, Swartz RH. Return to work after young stroke: a systematic review. Int J Stroke 2018; 13: 243–256. 29189108 10.1177/1747493017743059

[78] La Torre G, Lia L, Francavilla F, Chiappetta M, De Sio S. Factors that facilitate and hinder the return to work after stroke: an overview of systematic reviews. Med Lav 2022; 113: e2022029. 35766644 10.23749/mdl.v113i3.13238 PMC9437659

[79] Sandqvist JL, Björk MA, Gullberg MT, Henriksson CM, Gerdle BU. Construct validity of the Assessment of Work Performance (AWP). Work 2009; 32: 211–218. 19289874 10.3233/WOR-2009-0807

[80] Sargénius Landahl K, Sandqvist J, Bartfai A, Schult ML. Is a structured work task application for the assessment of work performance in a constructed environment, useful for patients with attention deficits? Diabil Rehabil 2021; 43: 1699–1709. 10.1080/09638288.2019.1674391 31642716

[81] Marom B, Jarus T, Josman N. The relationship between the Assessment of Motor and Process Skills (AMPS) and the Large Allen Cognitive Level (LACL) test in clients with stroke. Phys Occup Ther Geriat 2006; 24: 33–50.

[82] Sansonetti D, Hoffmann T. Cognitive assessment across the continuum of care: the importance of occupational performance-based assessment for individuals post-stroke and traumatic brain injury. Aust Occup Ther J 2013; 60: 334–342. 24089985 10.1111/1440-1630.12069

[83] Melby-Lervåg M, Hulme C. Is working memory training effective? A meta-analytic review. Dev Psychol 2013; 49: 270–291. 22612437 10.1037/a0028228

[84] Radomski MV, Anheluk M, Bartzen MP, Zola J. Effectiveness of interventions to address cognitive impairments and improve occupational performance after traumatic brain injury: a systematic review. Am J Occup Ther 2016; 70: 7003180050p1-9. 10.5014/ajot.2016.020776 27089289

[85] Sigmundsdottir L, Longley WA, Tate RL. Computerised cognitive training in acquired brain injury: a systematic review of outcomes using the International Classification of Functioning (ICF). Neuropsychol Rehabil 2016; 26: 673–741. 26965034 10.1080/09602011.2016.1140657

[86] Eriksson G. Occupational Gaps Questionnaire (OGQ). Swedish Association of Occupational Therapists 2017, version 2.0, Nacka, Sweden (in Swedish).

