# Supplementary material for: REHABILITATION WITH INTENSIVE ATTENTION TRAINING EARLY AFTER ACQUIRED BRAIN INJURY PROMOTES BETTER LONG-TERM STATUS ON HEALTH-RELATED QUALITY OF LIFE, DAILY ACTIVITIES, WORK ABILITY AND RETURN TO WORK
Source: J Rehabil Med. 2024 Jan 12;56:5308. doi: 10.2340/jrm.v56.5308 (PMC10802788; doi:10.2340/jrm.v56.5308)
Supplement: Supplementary file 1 — REHABILITATION WITH INTENSIVE ATTENTION TRAINING EARLY AFTER ACQUIRED BRAIN INJURY PROMOTES BETTER LONG-TERM STATUS ON HEALTH-RELATED QUALITY OF LIFE, DAILY ACTIVITIES, WORK ABILITY AND RETURN TO WORK [file JRM-56-5308-s1.pdf]

Supplementary material has been published as submitted. It has not been copyedited, typeset or checked for scientific content by Journal of Rehabilitation Medicine

Appendix S1. Distribution of activities examined in the Occupational Gaps Questionnaire and reported occupational gaps (limitations in activity) in four different domains of activities/participations. Distribution is presented in percentage in order of frequency of all patients (n=100)

**Instrumental ADL**		**Leisure activities**		**Social activities**		**Work/work related activities**	
Activity	*(%)*	*Activity*	*(%)*	*Activity*	*(%)*	*Activity*	*(%)*
Cleaning	32	Cultural activities	24	Travelling for pleasure	28	Working	37
Performing heavy maintenance	25	Participating in sports	22	Engaging in non-profit organizations	20	Studying	33
Laundry	23	Hobbies	22	Visiting relatives/friends	12	Performing voluntary work	20
Cooking	14	Reading books or periodicals	22	Visiting restaurants and bars	12	Raising children	7
Performing light maintenance	14	Reading newspapers	18	Visiting partner/children	10		
Personal finance	9	Writing	17	Participating in religious activities	6		
Grocery shopping	8	Outdoor life	16				
Transportation	1	Playing boardgames, etc	9				
		TV/video/radio	2				
		Using the computer	2				
